# Morphological characterization of NG2 glia and their association with neuroglial cells in the 3-nitropropionic acid–lesioned striatum of rat

**DOI:** 10.1038/s41598-018-24385-0

**Published:** 2018-04-13

**Authors:** Xuyan Jin, Tae-Ryong Riew, Hong Lim Kim, Jeong-Heon Choi, Mun-Yong Lee

**Affiliations:** 1 Department of Anatomy, Catholic Neuroscience Institute, College of Medicine, The Catholic University of Korea, Seoul, Korea; 2 Integrative Research Support Center, Laboratory of Electron Microscope, College of Medicine, The Catholic University of Korea, Seoul, Korea

## Abstract

Our aim was to examine the spatiotemporal profiles and phenotypic characteristics of neuron-glia antigen 2 (NG2) glia and their associations with neuroglial cells in striatal lesions due to the mitochondrial toxin 3-nitropropionic acid (3-NP). In control striatum, weak NG2 immunoreactivity was restricted to resting NG2 glia with thin processes, but prominent NG2 expression was noted on activated microglia/macrophages, and reactive NG2 glia in the lesion core after 3-NP injection. Activation of NG2 glia, including enhanced proliferation and morphological changes, had a close spatiotemporal relationship with infiltration of activated microglia into the lesion core. Thick and highly branched processes of reactive NG2 glia formed a cellular network in the astrocyte-free lesion core and primarily surrounded developing cavities 2–4 weeks post-lesion. NG2 glia became associated with astrocytes in the lesion core and the border of cavities over the chronic interval of 4–8 weeks. Immunoelectron microscopy indicated that reactive NG2 glia had large euchromatic nuclei with prominent nucleoli and thick and branched processes that ramified distally. Thus, our data provide detailed information regarding the morphologies of NG2 glia in the lesion core, and support the link between transformation of NG2 glia to the reactive form and microglial activation/recruitment in response to brain insults.

## Introduction

Neuron-glia antigen 2 (NG2) glia are characterized by expression of surface chondroitin sulfate proteoglycan 4^1–5^. NG2 glia were first described as progenitors for oligodendrocytes^6^. However, they have been recognized as a fourth neuroglial cell type in the mammalian central nervous system (CNS) and are distinct from astrocytes, mature oligodendrocytes, and microglia^7–10^. NG2 glia retain their proliferative ability throughout life. In fact, new oligodendrocytes and myelin continue to be produced even in the mature CNS, although the proliferation rate of NG2 glia peaks during the perinatal period^11–15^. Despite the even distribution of NG2 glia within all areas of the developing and mature CNS, NG2 glia comprise a highly heterogeneous population, and have diverse properties and functions^10,16^. In particular, a recent study demonstrated that NG2 glia are involved in the maintenance of neuronal function and survival through the regulation of neuroimmunological functions in the mature CNS^17^. These features suggest a complex role of NG2 glia in the CNS rather than a function solely as progenitors for oligodendrocytes.

In addition to the established role of NG2 glia in the developing and adult CNS, recent studies have revealed that NG2 glia play critical roles in various pathological conditions. It is widely accepted that NG2 glia proliferate and differentiate into myelinating oligodendrocytes and thereby repair the brain in demyelinating disease^10,13,18–21^. In addition to demyelination, NG2 glia undergo proliferation and morphological changes in response to various acute CNS insults, including stab wounds^22,23^, ischemia^24^, and spinal cord injury^19^, although the nature and time course of the appearance of reactive NG2 glia varies depending on the nature of the insult. Furthermore, NG2 glia are closely related to other two glial cell types, namely astrocytes and microglia, in the injured CNS^25–28^.

In addition to constitutive NG2 glia, activated microglia and infiltrated macrophages express NG2 following various CNS insults^29–35^. NG2 is also expressed in vascular mural cells, wherein it is upregulated during structural remodeling under pathological conditions^36^. Although there is substantial information regarding these heterogeneous populations of NG2-expressing cells in the injured CNS, their precise phenotypes, morphological characteristics, and temporal regulation patterns after insults remain to be established.

In this study, we examined the time course and distribution of and the cell types involved in the induction of NG2 expression in the lesioned striatum during the 8 weeks following an injection of the natural mitochondrial toxin 3-nitropropionic acid (3-NP). The mycotoxin 3-NP selectively damages medium spiny neurons in the striatum via several mechanisms involving excitotoxicity and oxidative stress^37,38^. This experimental model has advantages in the study of a series of pathophysiological responses, including changes in cellular dynamics and interactions among neuroglial cells in response to acute brain insults. This is because it leads to the formation of tissue lesions consisting of well-demarcated lesion cores where cell death occurs in neurons and in neuroglial cells, including astrocytes, microglia, and oligodendrocytes. Moreover, perilesional areas with astroglial hypertrophy and resultant astroglial scar formation can be observed^39–42^. We focused our attention on the morphological characteristics of NG2 glia and the interactions among NG2 glia, astrocytes, and microglia. We used double- and triple-labelling techniques to investigate various cell type-specific markers. In addition, the combined use of an immunoperoxidase method and a correlative approach using light and electron microscopy provided detailed and precise information regarding our ultrastructural observations in heterogeneous populations of NG2-expressing cells.

## Results

### Analysis of neurodegeneration in striata subjected to 3-NP treatment

As previously reported^43^, rats intoxicated with 3-NP developed characteristic neurological deficits, which were referred to as stage I, stage II, or stage III. About 70% of all experimental animals in our study progressed to stage III, and had hindlimb impairment, recumbency, and impaired postural adjustments. Experimental rats had reproducible and well-demarcated lesions confined to the lateral part of the striatum, although the sizes and extents of the lesions were somewhat different in each animal. The spatiotemporal regulation of neuroglial cells in the striatal lesions was equivalent in all animals.

Neurodegeneration after 3-NP injection was assessed using neuronal nuclear marker (NeuN) and terminal deoxynucleotidyl transferase dUTP nick end labelling (TUNEL) double staining. No specific TUNEL staining was detected in NeuN-expressing striatal neurons from control rats (Suppl Fig. 1a). In rats subjected to 3-NP treatment, TUNEL staining was evident in a few neurons in the lateral part of the striatum six hours post-lesion (Suppl Fig. 1b,c). One day after 3-NP injection, the number of TUNEL-positive cells in the lesion core, most of which were neurons, appeared to be higher than that noted at six hours post-lesion (Suppl Fig. 1d–f). On day 2, TUNEL staining was observed in most of the remaining striatal neurons, which were weakly labeled with NeuN (Suppl Fig. 1g–i). By days 3 (Suppl Fig. 1j–l) and 7 (Suppl Fig. 1m–o), almost all striatal neurons had virtually disappeared from the lesion core, although some neuronal debris remained.

### Spatiotemporal expression of NG2-positive cells in striata subjected to 3-NP treatment

**Figure 1:**
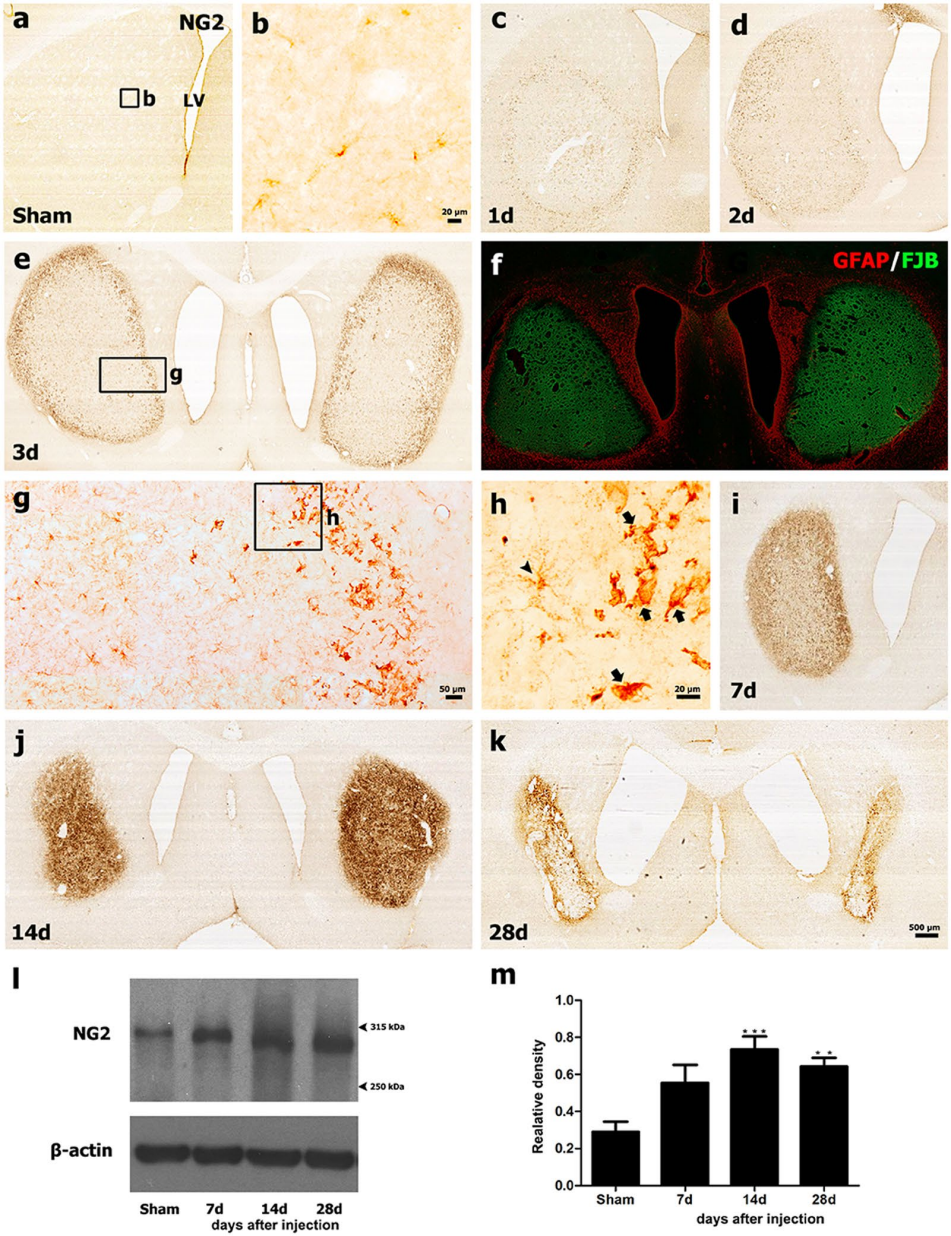
Representative images showing the temporal profiles of NG2-positive cells in 3-NP-treated rat forebrains. (**a**) Low-magnification view of a coronal section from a saline-treated control. (**b**) Higher-magnification view of the boxed area shown in a. Note that weak NG2 expression was observed in small stellate cells with fine processes in control striatum. (**c**–**e**) Prominent NG2 immunoreactivity appeared at the lesion edge by day 1 (**c**), and appeared to move into the epicenter on days 2 (**d**) and 3 (**e**) post-lesion. The boxed areas in e and g are enlarged in g and h, respectively. Note that NG2-positive cells in the lesion periphery had irregular cell bodies with thick and short processes (arrows in h), while small stellate cells with long fine processes (arrowhead in h) were preferentially found in the epicenter. (**f**) Merged image of FJB staining and GFAP immunoreactivity using serial striatal sections obtained 3 days post-lesion. Note that the lesion core is distinguished by intense FJB staining and concomitant loss of GFAP-positive astrocytes. (**i**–**k**) Changes in NG2 immunoreactivity in striatal sections from 3-NP-treated rats 7 (**i**), 14 (**j**), and 28 days (k) post-lesion. Prominent NG2 immunoreactivity was localized within both the lesion edge and the epicenter. LV: lateral ventricle. (**l**) Representative western blot analysis results for NG2 protein expression in striatal extracts from sham controls and rats killed 7, 14, and 28 days post-lesion. Note that a band of about 300 kDa corresponding to NG2 protein was clearly observed in both the control and lesioned striatum. (**m**) Quantification of NG2 protein expression. Data were obtained using densitometry and were normalized using β-actin as the loading control. The results are expressed in relative optical density and represent means ± SEM. The intensity of NG2 protein reactivity significantly increased by 14 days after 3-NP injection and then decreased, although enhanced expression levels persisted until at least day 28. **P < 0.01; and ***P < 0.001 vs. sham.

We first examined the spatiotemporal distribution of NG2-positive cells in rat striata subjected to 3-NP using immunohistochemistry. An example of the time course of the effects of 3-NP on the expression of NG2 is depicted in Fig. 1. Weak immunoreactivity for NG2 was detected in small stellate cells with fine processes in the cortices and striata of saline-treated control rats (Fig. 1a,b) and in the un-lesioned cortices of rats injected with 3-NP (data not shown). One day after 3-NP injection (Fig. 1c), prominent NG2 immunoreactivity began to appear at the edge of the lesion core. This distribution pattern was more pronounced on day 2 (Fig. 1d). Three days post-lesion, NG2 immunoreactivity was more prominent in the lesion edge and appeared to be moving into the epicenter (Fig. 1e). As shown in higher magnification images (Fig. 1g,h), NG2-positive cells with irregular cell bodies and thick and short processes were confined to the periphery of the lesion core, while small stellate cells with fine processes were mainly localized centrally. At this time point, the lesion core was examined by staining using Fluoro-Jade B (FJB), which is a fluorescent dye that labels degenerating neurons, and by assessing the loss of immunoreactivity for the astroglial marker glial fibrillary acidic protein (GFAP) (Fig. 1f), as described previously^40,41,44^. On day 7 (Fig. 1i) post-injection, NG2 immunoreactivity was observed within all regions of the lesion core, including the edge and the center, although it seemed to be more densely distributed at the lesion edge. This expression pattern was maintained on day 14, although intense NG2 immunoreactivity was evenly distributed throughout the lesion core (Fig. 1j). Twenty-eight days after the 3-NP injection, NG2 immunoreactivity appeared to be preferentially localized at the periphery of the striatal lesion in affected animals (Fig. 1k). The expression profile of NG2 was also examined using immunoblot analysis of protein extracts from the striatum of control and experimental rats. Immunoblotting revealed one band of about 300 kDa corresponding to NG2 protein in both the control and lesioned striata (Fig. 1l). The intensity of NG2 expression increased in the lesioned striatum by 14 days after 3-NP administration, and then decreased, although enhanced expression levels were maintained until at least day 28, which was the latest time point examined (Fig. 1m).

### Spatiotemporal profiles and relationships among NG2 glia, astrocytes, and microglia in the striatum in the early phase following 3-NP injection

**Figure 2:**
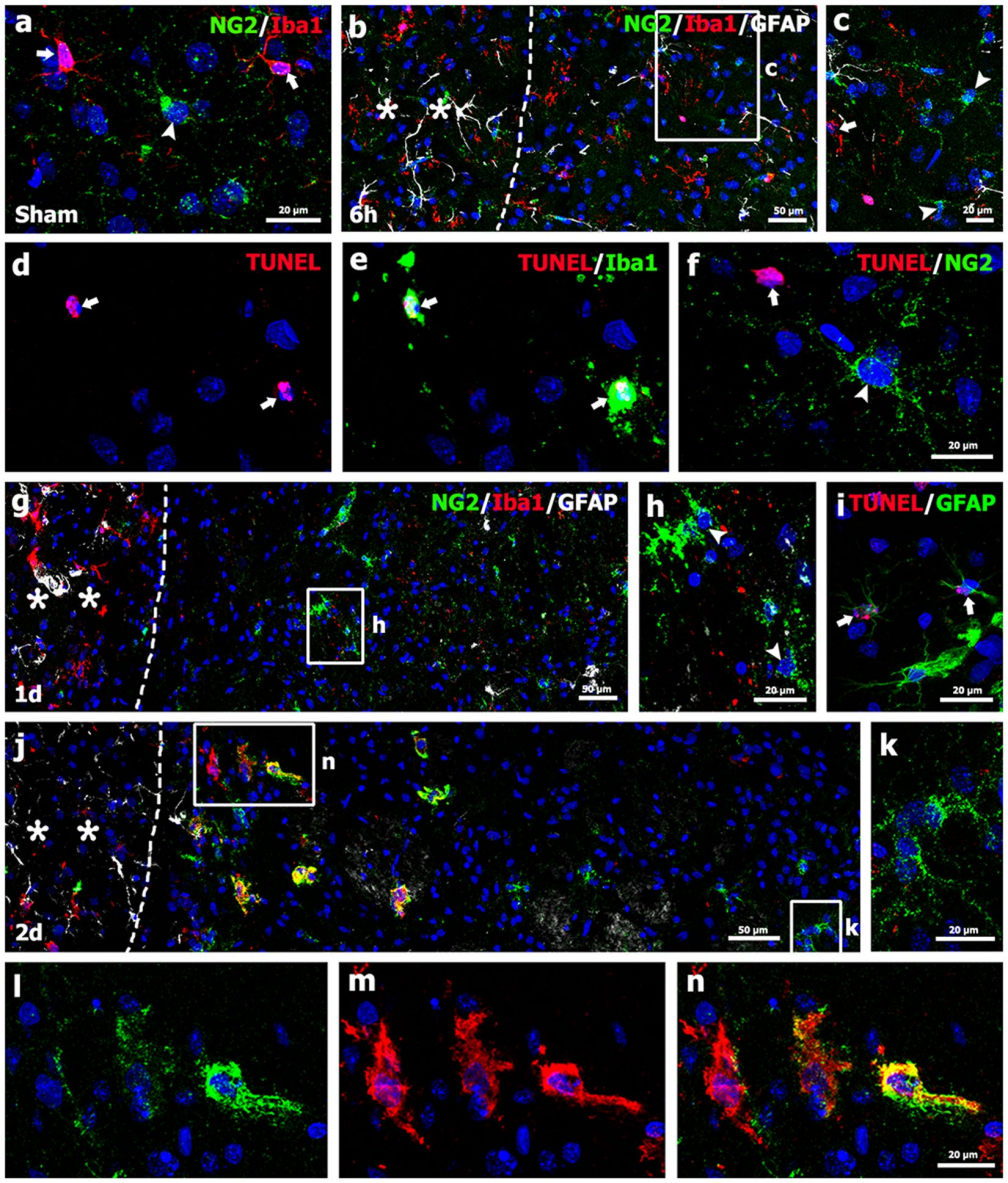
Spatial relationships between NG2-positive cells and neuroglial cells in control sections and in the lesion core 6 hours and 1 and 2 days after 3-NP injection. (**a**) In control sections, NG2-positive cells (arrowhead) and Iba1-positive microglia (arrows) did not have overlapping distributions. (**b**) Triple-labeling for NG2, Iba1, and GFAP six hours post-lesion revealed a well-demarcated lesion core (right side of the broken line), which was clearly distinguishable from the peri-lesional area (asterisks) by prominent decrease of GFAP immunoreactivity. (**c**) Higher-magnification views of the boxed area in b showing that microglia displayed irregular and fragmented morphologies (arrow), while NG2-positive cells had morphologies similar to those in control (arrowheads). (**d**,**e**) Double-labeling for Iba1 and TUNEL indicated that Iba1-positive microglia were dying cells (arrows in **d** and **e**), (**f**) Double-labeling for TUNEL and NG2 indicated that the TUNEL-positive cell (arrow) was not positive for NG2, or vice versa (arrowhead). (**g**,**h**) Triple-labeling for NG2, Iba1, and GFAP one day post-lesion indicated that NG2-positive cells with multiple processes (arrowheads in h) were observed in the lesion core (right side of the broken line), where GFAP and Iba1 staining were very weak or negligible. Note that astrocytes and microglia were observed in the peri-lesional area (asterisks in **g**). The boxed area in g is enlarged in h. (**i**) Double-labeling for GFAP and TUNEL indicated that some remaining astrocytes (arrows) in the lesion core were positive for TUNEL. (**j**) Triple-labeling for NG2, Iba1, and GFAP two days post-lesion showing that NG2 expression appeared to overlap with Iba1 expression at the lesion edge, but not in the epicenter, both of which were devoid of GFAP immunoreactivity. The broken line indicates the border between the lesion core and the peri-lesional area (asterisks). (**k**–**n**) Higher-magnification views of the boxed areas in j. (**k**) NG2-positive cells with radially oriented thin processes were observed in the epicenter, where Iba1-positive microglia were absent. (**l**–**n**) Note that NG2 expression was observed on the surface or within the cytoplasm of activated microglia, and that NG2 expression tended to increase from the lesion edge to the epicenter. Cell nuclei were stained with DAPI.

As shown in Fig. 1, the distribution pattern of NG2 immunoreactivity in the lesion core changed during the post-injury period. We first performed double-labeling experiments for NG2 and Iba1, which is a well-established marker for microglia. NG2-positive cells with thin processes and resting microglia with ramified morphology were identified in the striata of control-treated rats. However, the distributions of these cell types did not overlap (Fig. 2a). For the detailed characterization of the NG2-positive cells and their association with neuroglial responses in the lesion core following 3-NP injection, we performed triple-labeling for NG2, and Iba1 and GFAP, which are well-established markers of microglia and astrocytes, respectively. Six hours after 3-NP injection, a well-demarcated lesion core was evident in the lateral part of the striatum, and a prominent decrease in GFAP-positive astrocytes with normal morphology was observed (Fig. 2b). In the lesion core, Iba1-positive microglia displayed irregular and shrunken morphologies with fragmented processes, whereas NG2-positive cells showed morphologies similar to those observed in neurons found in the control striatum (Fig. 2b,c). Thus, these data indicate that most microglia in the lesion core were dying or dead as early as 6 hours post-injection.

Triple-labeling for NG2, Iba1, and GFAP one day after 3-NP injection revealed prominent NG2 immunoreactivity in cells with evident processes in the lesion core, where a loss of astrocytes and microglia with normal morphology was observed. However, both glial cell types were present in the peri-lesional area (Fig. 2g). At higher magnification (Fig. 2h), it was clear that NG2-positive cells had similar morphological features to resting NG2 glia, which had small cell bodies from which multiple fine processes radiated in all directions^45–47^. At this time point, double-labeling for GFAP and TUNEL staining revealed that some remaining astrocytes in the lesion core were positive for TUNEL (Fig. 2i).

On day 2 after 3-NP injection, triple-labeling for NG2, Iba1, and GFAP revealed that NG2 expression appeared to overlap with Iba1 expression at the lesion edge, which was devoid of GFAP immunoreactivity (Fig. 2j). Interestingly, NG2 expression was localized to the surface and cytoplasm of activated microglia, whose NG2 expression levels had a tendency to increase in prominence when moving from the lesion edge to the epicenter (Fig. 2l–n). In contrast, no Iba1-positive microglia were observed within the epicenter of the lesion core, where ramified NG2 glia with thin processes were present (Fig. 2k).

**Figure 3:**
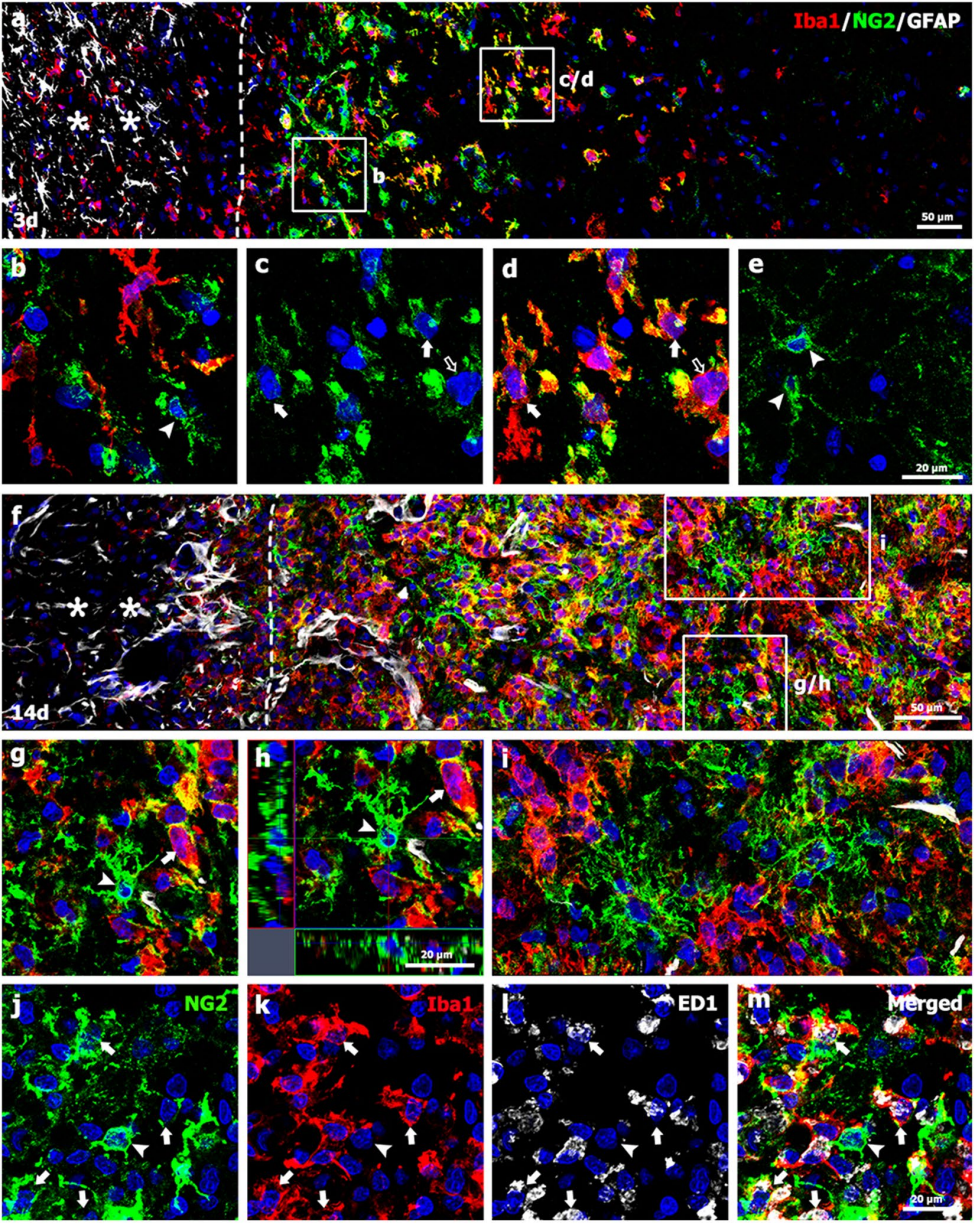
Characterization of NG2-positive cells and their spatial relationships with neuroglial cells in the lesion core on days 3 and 14 post-lesion. (**a**) Triple-labeling for NG2, Iba1, and GFAP showing that Iba1-positive microglia and NG2-positive cells were preferentially localized to the periphery of the lesion core (right side of the broken line), but not in the peri-lesional area (asterisks). Both the periphery and epicenter were devoid of GFAP immunoreactivity. (**b**–**d**) Higher-magnification views of the boxed areas in a. Note that NG2 expression was observed in reactive NG2 glia with thick processes (arrowhead in b) and some activated microglia/macrophages, i.e., activated stellate microglial cells with thick and short processes (arrows in **c** and **d**), and amoeboid macrophages with retracted processes (open arrows in c and d). (**e**) Higher magnification image of the lesion epicenter showing ramified NG2 glia (arrowheads). (**f**) Triple-labeling for NG2, Iba1, and GFAP showing that the lesion cores (right side of the broken line) were filled with cells expressing NG2 or Iba1 14 days post-lesion. Asterisks in f point to the perilesional area. (**g**–**i**) Higher-magnification views of the boxed areas in f. Note that NG2 expression was localized to NG2 glia with thick and complexly branched cytoplasmic processes (arrowheads in g and h) and activated microglia (arrows in g and h). In addition, NG2 glia appeared to be entangled with each other (**i**). (**j**–**m**) Triple-labeling with NG2, Iba1, and ED1 shows that NG2 expression was localized to most Iba1^+^/ED1^+^ cells with morphologies reminiscent of amoeboid brain macrophages (arrows in j–m). Arrowheads in j–m point to NG2 glia. Cell nuclei were stained with DAPI.

On day 3 after 3-NP injection, triple-labeling for NG2, Iba1, and GFAP revealed that NG2 and Iba1 were preferentially localized in the lesion periphery, but appeared to infiltrate into the epicenter (Fig. 3a). The lesion core was devoid of GFAP immunoreactivity. In the lesion periphery, NG2-positive cells could be divided into two groups according to co-expression with Iba1, namely, NG2^+^/Iba1^−^ cells and NG2^+^/Iba1^+^ cells (Fig. 3a). Among these cells, NG2^+^/Iba1^−^ cells had morphologies similar to reactive NG2 glia with larger cell bodies and short and thick processes (Fig. 3b), as reported previously^45,48^. In addition, NG2^+^/Iba1^+^ cells comprised a subpopulation of activated microglia/macrophages, i.e., activated stellate microglial cells with thick and short processes, and amoeboid brain macrophages with retracted processes (Fig. 3c,d). In contrast, NG2-positive cells in the lesion epicenter, where microglia had not yet infiltrated, appeared as ramified NG2 glia (Fig. 3e).

Fourteen days post-lesion, the numbers and staining intensities of NG2-positive cells appear to increase in the lesion core when compared to the 3-day time point (Fig. 3f). At this time point, NG2 glia exhibited distinctive morphologies with thick processes with complex branching patterns (Fig. 3g,h). These processes appeared to be entangled with each other, often in extensive areas of the lesion core (Fig. 3i). In addition, NG2 expression was observed in a subset of Iba1-positive microglia/macrophages (Fig. 3g–i). We further characterized these NG2-expressing microglia/macrophages via triple-labeling with NG2, Iba1, and ED1, which is a marker for the lysosomal membranes of macrophages^49^. Most Iba1^+^/ED1^+^ cells, which were large and round, had fewer shorter processes, and resembled amoeboid brain macrophages, had NG2 immunoreactivity (Fig. 3j–m).

**Figure 4:**
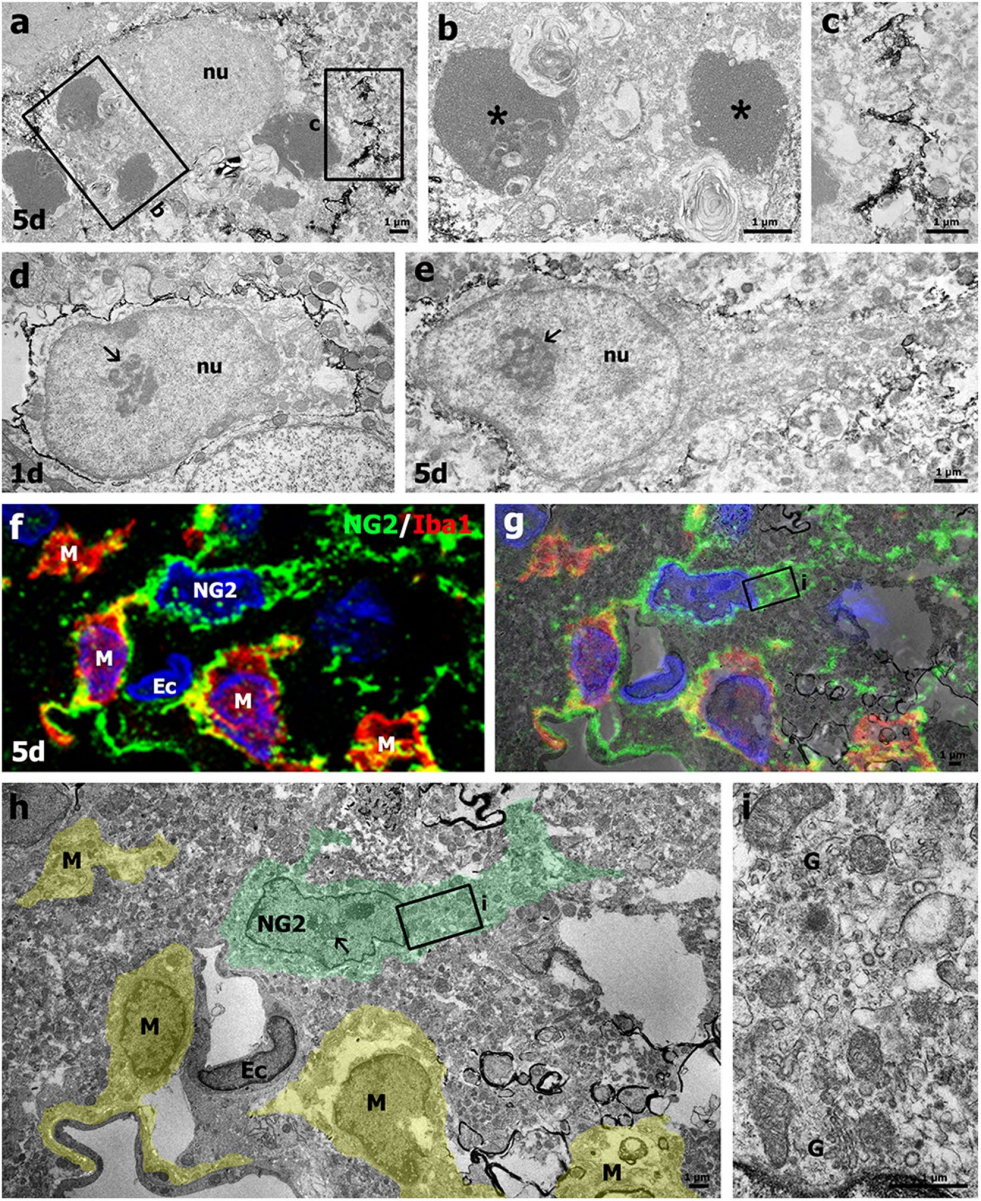
Ultrastructural characterizations of NG2-positive cells in the lesion core. (**a**–**c**) Immunoelectron microscopic images of the lesion core 5 days post-lesion show that NG2 expression was specifically localized along the plasma membrane of brain macrophages and the adjacent extracellular matrix. (**b**,**c**) Higher-magnification views of the boxed areas in a. Asterisks in b denote well-developed phagolysosomal structures. (**d**,**e**) Ultrastructural characterization of NG2 glia in the lesion core on days 1 and 5 post-lesion. NG2 expression was localized along the plasma membranes of cells with large euchromatic nuclei (nu) with prominent nucleoli (arrows in d and e) on days 1 (**d**) and 5 (**e**). Note that NG2-positive cells observed on day 5 post-lesion had more cytoplasm and thicker processes than those found on day 1. (**f**–**i**) Confocal microscopic image of a semi-thin section double-labeled with NG2 and Iba1 (**f**), and the corresponding transmission electron microscopic image (**h**) obtained from the same field within the striatal lesion 5 days post-lesion. (**g**) Overlay image of confocal microscopic data onto the corresponding electron microscopic image. (**i**) Higher-magnification views of the boxed areas in g and h. Note that NG2 glia (NG2) had large euchromatic nuclei with prominent nucleoli (arrow in h) and cytoplasm containing a well-developed Golgi complex (G in i), while NG2-positive microglia (M) had oval or triangular nuclei with dense and highly clumped heterochromatin. Ec: endothelial cell. Cell nuclei were stained with DAPI.

We further classified the NG2-positive cells into NG2 glia and NG2-positive microglia based on immunoelectron microscopic observations. As shown in Fig. 4a–c, electron-dense NG2 immunostaining was specifically observed along the plasma membrane and the adjacent extracellular matrix of brain macrophages. The stained cells displayed prominent features of phagocytic activity, such as well-developed phagolysosomal structures. NG2 immunostaining was also detected on the plasma membranes of cells presumed to be NG2 glia, which were characterized by large euchromatic nuclei with prominent nucleoli (Fig. 4d,e). These cells had scarce cytoplasmic organelles on day 1 post-lesion (Fig. 4d), but they were enlarged and had expanded cytoplasm and broad processes on day 5 (Fig. 4e). We used a correlative light- and electron-microscopic approach to determine whether these cells were indeed NG2 glia. Semi-thin sections double-labeled with NG2 and Iba1 were first observed using confocal microscopy, which clearly revealed two types of NG2-positive cells, activated microglia and NG2 glia devoid of Iba1 immunoreactivity (Fig. 4f). The same semi-thin sections were subsequently processed further for examination using electron microscopy (Fig. 4h). The confocal microscopy and transmission electron microscopy data combined in overlay confirmed that NG2 glia had large euchromatic nuclei with prominent nucleoli and well-developed Golgi complexes, while NG2-positive microglia had oval or triangular nuclei with dense and highly clumped heterochromatin (Fig. 4g–i).

### Quantitative time-dependent analysis of NG2 glia and NG2-positive microglia in striata subjected to 3-NP treatment

**Figure 5:**
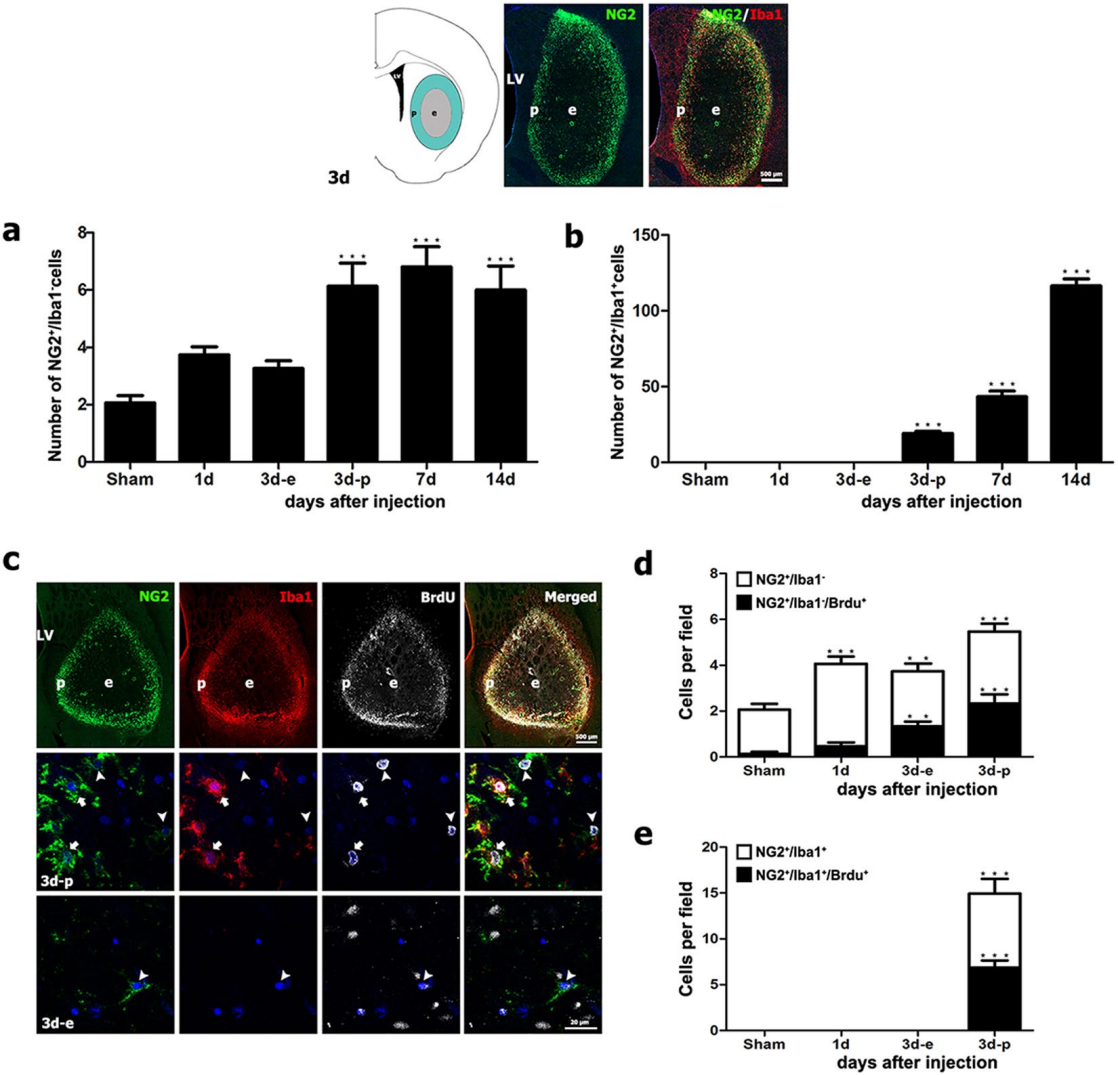
Quantitative analysis of the numbers and proliferation of NG2 glia (NG2^+^/Iba1^−^ cells) and NG2-positive microglia (NG2^+^/Iba1^+^ cells) in the lesion core in 3-NP-treated rats. Three days post-lesion, the lesion core can be divided into two areas: the epicenter (**e**), which is devoid of Iba1-positive microglia, and the lesion periphery (p), which is heavily infiltrated by activated microglia (see Fig. 1e). (**a**,**b**) Note that the numbers of NG2 glia increased progressively and reached a maximum 7 days post-lesion. They then decreased slightly on day 14 (**a**). The numbers of NG2-positive microglia increased progressively until day 14 (**b**). The data are expressed as the mean ± SEM. ***P < 0.001 vs. sham. (**c**) Triple-labeling for NG2, Iba1, and BrdU 3 days post-lesion. (Upper row) NG2 and Iba1 were preferentially localized to the lesion periphery (p) when compared to the epicenter (**e**). This was similar to the distribution pattern of BrdU-positive cells. Higher-magnification views of the lesion periphery (middle row) and epicenter (lower row) showing that NG2 glia (arrowheads in middle row) and NG2-positive microglia (arrows in middle row) in the lesion periphery frequently had BrdU labeling, while some NG2 glia (arrowheads in lower row) were positive for BrdU in the microglia-free epicenter. (**d**) Absolute numbers of NG2 glia and BrdU-labeled NG2 glia in control striatum and in the lesion core in 3-NP-treated rats 1 and 3 days post-lesion. Note that at the numbers of proliferating NG2 glia were higher in the lesion edge when compared to the epicenter 3 days post-lesion. (**e**) Absolute numbers of NG2/Iba1/BrdU triple-labeled cells and NG2-Iba1 double-labeled cells in control striatum and in the lesion core 1 and 3 days post-lesion. Note that proliferating NG2-positive microglia comprised 46.0% of all NG2-positive microglia in the lesion periphery 3 days post-lesion. The data are expressed as the mean ± SEM. **P < 0.01 and ***P < 0.001 vs. sham.

As described above, the numbers of NG2-positive cells appeared to increase in the lesion core over a 14-day period post-injection. Quantification of NG2 glia and NG2-positive microglia in the lesion core after 3-NP injection confirmed the visual inspection. The numbers of NG2 glia and NG2-positive microglia increased progressively until day 14 post-lesion, although the numbers of NG2 glia slightly decreased on day 14 (Fig. 5a,b). In particular, the numbers of NG2 glia in the lesion edge, where microglia had infiltrated, were significantly higher than in the microglia-free center in the striatum 3 days post-lesion. Thus, triple-labeling for NG2, Iba1, and 5-bromo-2′-deoxyuridine (BrdU) was performed to determine whether the increase in the numbers of NG2-positive cells in the lesion edge was due to new cells generated via proliferation. As shown in Fig. 5c, NG2 and Iba1 had overlapping regional distributions confined to the lesion periphery, where the proliferative cells that had incorporated BrdU were more abundant than in the epicenter 3 days post-lesion. Higher-magnification views revealed that NG2 glia and NG2-positive microglia frequently exhibited more BrdU labeling in the lesion periphery than in the microglia-free epicenter (Fig. 5c).

We quantified the numbers of NG2 glia and NG2-positive microglia proliferating in control striatum sections and in the lesion core on days 1 and 3 post-lesion. As shown in Fig. 5d, 6.2% and 11.5% of all NG2 glia were proliferating in control sections and in lesioned striata one day post-lesion, respectively. This proportion rose to 35.7% in the lesion edge and 42.6% in the epicenter 3 days post-lesion. However, the numbers of proliferating NG2 glia 3 days post-lesion were higher in the lesion periphery than in the epicenter. In addition, proliferating NG2-positive microglia comprised 46.0% of all NG2-positive microglia in the lesion edge 3 days post-lesion (Fig. 5e).

### Spatiotemporal profiles and relationships among NG2 glia, astrocytes, and microglia in striata subjected to 3-NP treatment in the chronic phase

**Figure 6:**
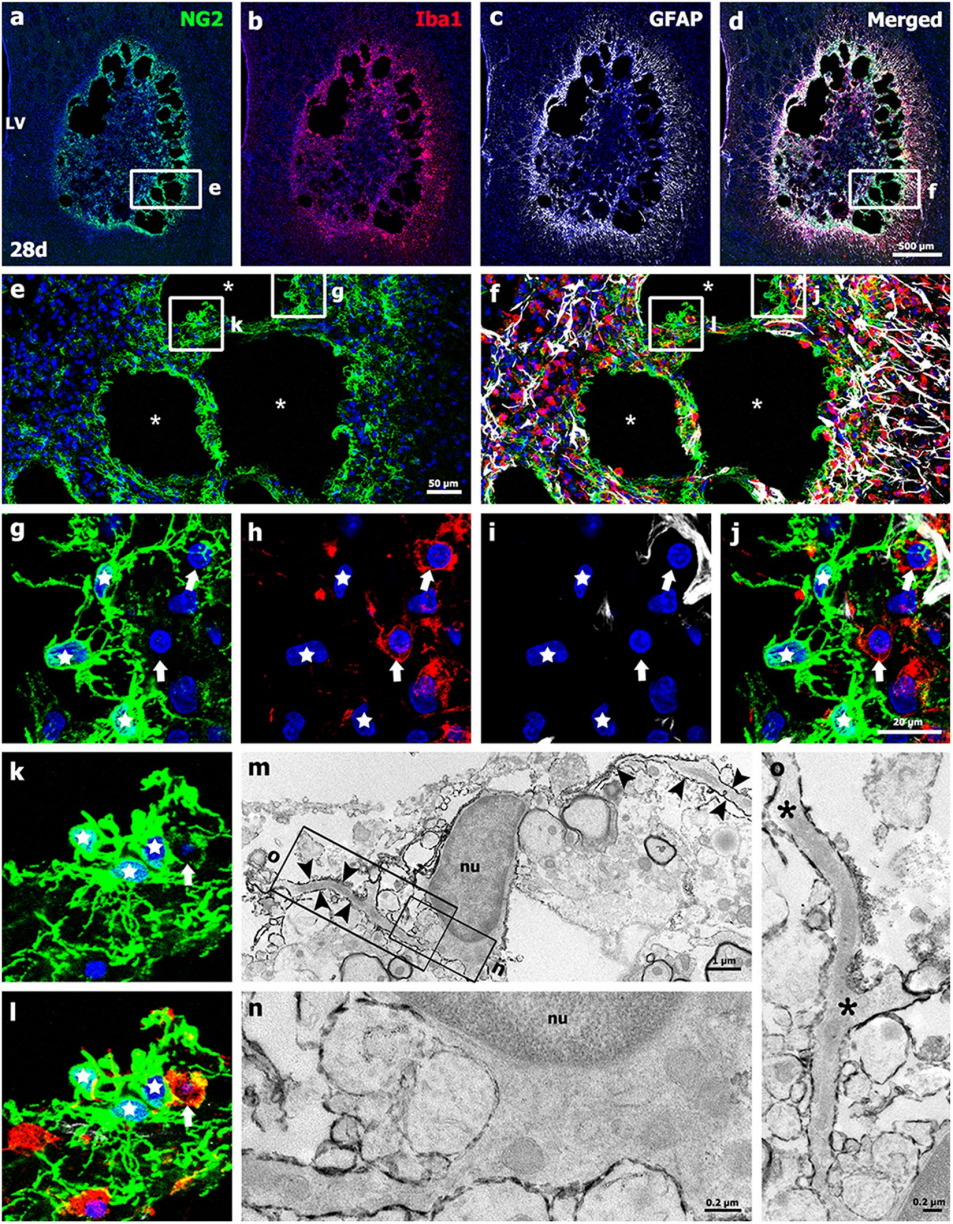
Characterization of NG2-positive cells and their spatial relationships with neuroglial cells in the lesion core on day 28 post-lesion. (**a**–**d**) Triple-labeling for NG2, Iba1, and GFAP revealed that immunoreactivities for NG2 and Iba1 were more prominent in the lesion periphery, where cystic cavitation is observed, than in the epicenter. A GFAP-positive astroglial scar was evident in the peri-lesional area. (**e**,**f**) Higher-magnification views of the boxed areas in a and d, respectively. Note that the cystic cavities (asterisks in e and f) were principally surrounded by NG2 glia. (**g**–**l**) Higher-magnification views of the boxed areas in e and f. Note that NG2 glia lining the cystic cavities had thick and complexly branched cytoplasmic processes and formed a dense meshwork. These cells were connected to neighboring NG2 glia (stars in **g**–**l**). In addition, NG2 expression in activated microglia/macrophages was still evident (arrows in **g**–**l**) at this time point. Cell nuclei were stained with DAPI. LV: lateral ventricle. (**m**–**o**) Ultrastructural characterization of NG2-positive cells in the lesion core on day 28 post-lesion. (**m**) NG2 staining was localized along the membrane of NG2 glia with euchromatic nuclei (nu) and several long processes (arrowheads in m). (n and o) Higher-magnification views of the boxed areas in m. Note that NG2-positive processes ramified distally (asterisks in o).

Triple-labeling for NG2, Iba1, and GFAP in the lesioned striatum 28 days post-lesion indicated that NG2 and Iba1 appeared to be preferentially localized to the periphery of the lesion core, which was surrounded by reactive astrocytes with distinctive elongated morphologies and long cellular processes (Fig. 6a–f). Higher-magnification images revealed that NG2 glia had thick and complexly branched cytoplasmic processes that were frequently in close apposition or intermingled with one another, forming a network in the lesion core (Fig. 6g–l). At this time point, multiple cystic cavities were often formed in the lesion periphery, and NG2 glia were preferentially localized in areas closely surrounding these cavities, with only sporadic astroglial processes (Fig. 6g–l). In contrast, microglial NG2 expression appeared to be considerably reduced when compared to 14 days post-lesion, with some microglia/macrophages expressing NG2 (Fig. 6f,h,j,l). Detailed ultrastructural investigation of NG2 glia in the striatal lesions in 3-NP-injected rats 28 days post-lesion indicated that electron-dense NG2 staining was localized along the plasma membranes of NG2 glia with euchromatic nuclei (Fig. 6m–o). These NG2 glia had several long cytoplasmic processes that ramified distally.

**Figure 7:**
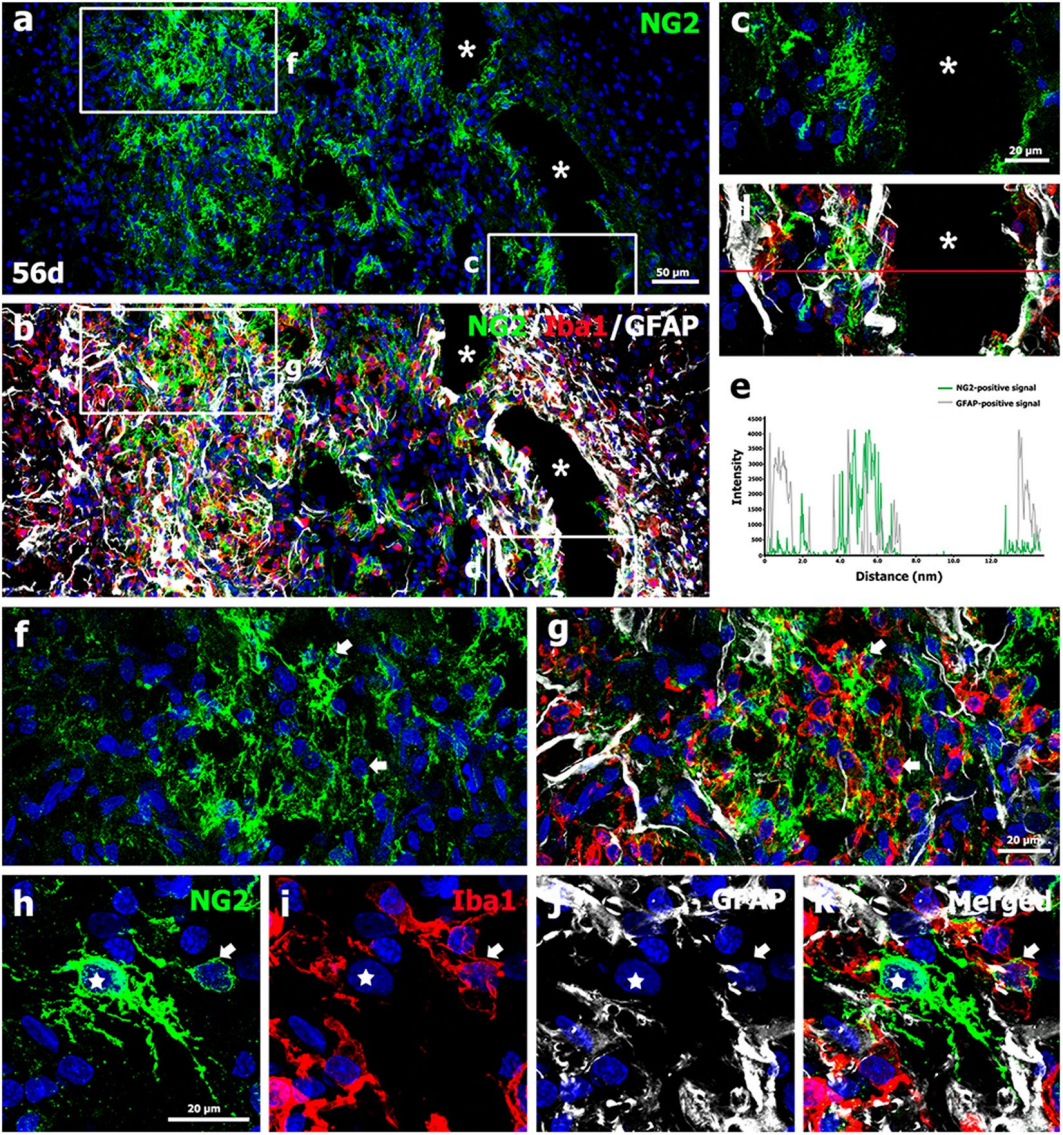
Characterization of NG2-positive cells and their spatial relationships with neuroglial cells in the lesion core on day 56 post-lesion. (**a**–**d**) Triple-labeling for NG2, Iba1, and GFAP indicated that NG2 expression was still confined to the lesion core, and in particular the periphery, where it colocalized with activated microglia/macrophages and reactive astrocytes. Note that NG2 expression was not observed in the astroglial scar bordering the lesion core. (**c**,**d**) Higher-magnification views of the boxed areas in a and b, respectively. Note that cavities (asterisks in **a**–**d**) were surrounded by a network mainly composed of NG2 glia and astroglial processes, which did not overlap. (**e**) Histogram of the intensity profiles of NG2-positive and GFAP-positive signals along the indicated area (red line) in d. Note that both signals had largely complementary patterns within the areas surrounding the cystic cavities. (**f**,**g**) Higher-magnification views of the boxed areas in a and b, respectively, showing that NG2 glia appeared to form a network in the lesion core. Note that some activated microglia/macrophage expressed NG2 (arrows in **f**,**g**), while most were devoid of NG2. (**h**–**k**) Higher-magnification views of the lesion core showing that NG2 expression was localized to NG2 glia with complex branching processes (stars in h–k) and to activated microglia/macrophages (arrows in h–k), but not to reactive astrocytes. Cell nuclei were stained with DAPI.

Fifty-six days post-lesion, NG2 immunoreactivity appeared to be localized in association with activated microglia/macrophages and GFAP-positive astrocytes in the lesion core, but was not detected in reactive astrocytes forming the glial scar in the peri-lesional area (Fig. 7a,b). As shown at higher magnification (Fig. 7c,d), cystic cavities in the lesion core were surrounded by a network mainly composed of NG2 glia and astroglial processes, both of which were closely associated with each other, but did not overlap. This finding was further supported by the intensity profiles of NG2-positive and GFAP-positive signals within the areas surrounding the cystic cavities, which revealed largely complementary patterns (Fig. 7e). At this time point, NG2 glia with complex branching processes appeared to form a network in the lesion core, while only some activated microglia/macrophages expressed NG2 (Fig. 7f–k).

### Expression of NG2 in the vascular structures in the striata of rats subjected to 3-NP treatment

In the striata of control rats, double-labeling for NG2 and the vascular endothelial cell marker RECA1 revealed weak NG2 expression in vascular cells, as well as in ramified NG2 glia (Suppl Fig. 2a,b). On day 1 post-lesion, double-labeling for NG2 and two vessel-associated markers, isolectin B4 (which labels vascular endothelial cells and activated microglia) and RECA1, revealed NG2 expression within or in close proximity to the vasculature (Suppl Fig. 2c,d). To more precisely characterize the vasculature-associated NG2-positive cells in the lesion core, pre-embedding immunoelectron microscopy was performed on brains obtained 1 and 5 days post-lesion. NG2 immunoreactivity, as indicated by highly electron-dense 3,3′-diaminobenzidine tetrahydrochloride grains, was specifically observed in the plasma membrane and the adjacent extracellular matrix of the pericytes (Suppl Fig. 2e,h,i) and smooth muscle cells (Suppl Fig. 2f–h,j), but not in endothelial cells. The plasma membrane caveolae of smooth muscle cells were highly immunoreactive for NG2 (Suppl Fig. 2f,g). In addition, electron-dense NG2 staining along the plasma membranes of smooth muscle cells in the striatal lesions in 3-NP-injected rats was maintained 28 days post-lesion (Suppl Fig. 2k).

## Discussion

We used 3-NP injection as a model to obtain detailed and precise information regarding the light microscopic and ultrastructural morphologies of NG2 glia and their relationships with other two glial cell types (astrocytes and microglia) in the lesioned striatum. Immunoblotting revealed the presence of a 300-kDa band in the protein extract from the striata of both control and lesioned rats, but the intensity of the band from the lesioned striatum extract was significantly higher. Our data is in contrast with that of previous studies on ischemic brains, wherein the molecular weight of NG2 in nonischemic regions was found to be 290 kDa, possibly owing to the post-translational modification of the NG2 core protein, i.e., the shedding of its extracellular domain^33,50^. Although the reason for this discrepancy is unclear, these previous studies did show that the expression of the dominant NG2 core protein with a molecular weight of 300 kDa was higher in the ischemic core region, which is in agreement with our results. We found that activation of NG2 glia was characterized by progressive morphological changes, higher proliferation rates, and more intensive immunostaining for the proteoglycan NG2. In addition, immunoelectron microscopy and a correlative approach using light and electron microscopy revealed that reactive NG2 glia had large euchromatic nuclei with prominent nucleoli and cytoplasms rich in organelles, and especially the Golgi complex. This implies the appearance of cells active in the synthesis and release of NG2. In particular, NG2 glia had characteristic thick and ramified processes at a later time point. Thus, our data reinforce the idea that NG2 glia may undergo proliferation and morphological changes in response to a wide variety of acute CNS insults^19,22–24^, and further provide a detailed morphological characterization of NG2 glia.

Our data clearly indicate that virtually all resident microglia died in the lesion core starting 6 hours to 1 day post-lesion, as suggested by the infarction core model of cerebral and spinal cord ischemia^51–53^. Instead of containing pre-existing microglia, the lesion core appeared to be progressively infiltrated with microglia migrating from the peri-lesional area. Activated microglia began to appear at the lesion edge starting on day 2 post-lesion, and were evenly distributed throughout the lesion core by day 14. The induction of NG2 expression in a subpopulation of the microglia in the lesion core was evident by 2 days post-lesion, and was still prominent 14 days after the induction of the lesion, although NG2 expression appeared to decrease over time. In agreement with previous reports of an NG2-positive zone delineating the infarct core from the peri-infarct zone after focal cerebral ischemia^50,54^, our data revealed that the characteristic NG2-positive zone was transiently observed at the edge of the lesion core. This zone was evident beginning one day after lesioning and became more prominent 3 days post-lesion. We confirmed that the presence of zone was indeed attributed to the accumulation of NG2 glia and NG2-positive microglia at the lesion edge. In addition, our data revealed that NG2/Iba1 double-labeled cells in the lesion core exhibited both macrophage-like phenotypes with amoeboid morphology and activated stellate microglial cells with evident processes, supporting the observations that NG2 expression is induced in bone marrow-derived monocytes/macrophages^29,30,33,34^ and activated microglia^31,55,56^ following injury to the CNS.

It is noteworthy that the activation of NG2 glia appeared to correlate temporally and spatially with infiltration of activated microglia into the lesion core. NG2 glia with distinct reactive morphologies began to appear at the lesion edge starting at 2 days post-lesion. These cells were evenly distributed throughout the lesion core by 14 days after lesion induction. In addition, the numbers of NG2 glia in the lesion edge were significantly higher than in the microglia-free epicenter 3 days post-lesion. This increase was attributed to enhanced proliferation of NG2 glia localized at the lesion edge, as determined using BrdU labeling experiments. Thus, our data indicated that in contrast to microglia, pre-existing NG2 glia can survive and transform into reactive cells. This transformation occurred in association with the activation and recruitment of microglia/macrophages in the lesion core over time. A close relationship between NG2 glia and activated microglia has previously been noted following several types of insults. This suggests the presence of functional and complex interactions between activated microglia/macrophages and NG2 glia^25,45,57^. Specifically, Schonberg, *et al*.^57^ have suggested that activated microglia/macrophages influence the proliferation of NG2 glia and the differentiation of oligodendrocytes after CNS trauma. Taken together, our data suggest that activation of NG2 glia may be due in part to many cytokines, chemokines, and other proteins secreted by activated microglia/macrophages in the injured CNS^28^, although the exact mechanism is still unknown.

While activation of NG2 glia in response to a wide variety of acute CNS insults is well established, the specific roles of these cells are still obscure. Reactive and proliferating NG2 glia residing at or close to demyelination lesions function as oligodendrocyte precursor cells^18,20,21,58^ or generate astrocytes after CNS insults^59–62^. However, remyelination by NG2 glia may not reach completion due to the absence of several factors required for remyelination, or due to the inhibitory lesion environment, as suggested previously^10,63^. In addition, several reports have provided evidence that reactive astrocytes within or in the vicinity of lesions are derived from pre-existing astrocytes, and not from NG2 glia^22,23,64^. However, other potential roles for NG2 glia in scar formation after CNS insults have been described. Hughes, *et al*.^65^ have demonstrated that CNS injury induces transformation of NG2 glia into distinct scar-forming cells with unique characteristics. This suggests that enhanced proliferation of NG2 glia contributes to glial scar formation following several types of CNS trauma. In addition, NG2 glia and microglia are the first cells to react and strongly accumulate around the injury site, while no astrocytes actively migrate toward the injury site after brain insults^66^. This implicates NG2 glia and microglia in functions previously thought to be carried out by astrocytes^10,22,24,30,67^. Moreover, NG2 glia and astrocytes are intimately associated and their cell bodies and processes are extensively interwoven^68^. In addition, NG2 glia and astrocytes are major components of the glial scar after CNS insults^30,55,61^. It is thus interesting that the morphologies of NG2 glia were dramatically altered with time. Specifically, the thin and unbranched processes of these cells were transformed into thicker and highly ramified processes forming a dense network in the astrocyte-free lesion core by 2 weeks after the injury. NG2 glia then gradually became closely associated with reactive astrocytes and formed a cell network in the lesion core over a period of 4–8 weeks. Thus, the aforementioned studies and our data suggest that NG2 glia may be the primary cells involved in fibrotic scar formation during the early stages of injury.

Interestingly, our data indicate that developing cavities, which occurred secondary to initial traumatic injury, were first surrounded by reactive NG2 glia 4 weeks after injury and by a network mainly composed of NG2 glia and astroglial processes 8 weeks after injury. This suggests that the scar tissue encapsulating cavities was mainly derived from NG2 glia in the early phase, and later included both reactive astrocytes and NG2 glia. Consistent with our findings, Fitch and Silver^69^ reported that increased levels of chondroitin sulfate proteoglycans are localized to the edges of developing cavities in the injured CNS and are surrounded by astrocytes in the tissue parenchyma. This suggests that proteoglycans secreted by reactive astrocytes may protect the surrounding tissue from the injured tissue. In addition, Fitch, *et al*.^70^ have suggested that proteoglycans surrounding the cavities may be involved in astrocyte migration during cavitation. This hypothesis is based on previous studies implicating proteoglycans in cell motility and migration^71,72^. Here we extend this concept by showing that the glial scar tissue surrounding the cavities was comprised primarily of NG2 glia before the involvement of astrocytes. Our findings suggest that NG2 glia may protect the surrounding tissue by encapsulating the cavities and that they may be involved in astrocyte migration during cavitation.

NG2 expression was also noted in vasculature-associated cells, including pericytes and smooth muscle cells in the lesion core. This was confirmed using immune-electron microscopy. NG2 expression in these vascular cells was prominent within the first few days after 3-NP injection and was sustained over a 4-week period. Several studies indicate that NG2 expression in pericytes and smooth muscle cells is upregulated during vascular remodeling and morphogenesis. This suggests that NG2 is involved in stimulating pericyte proliferation and motility and recruiting endothelial cells^36,73–76^. Thus, our data indicate that NG2 expression in pericytes and smooth muscle cells in the lesion core may contribute to their angiogenic potential, although the precise role of NG2 in this process remains to be elucidated.

**Figure 8:**
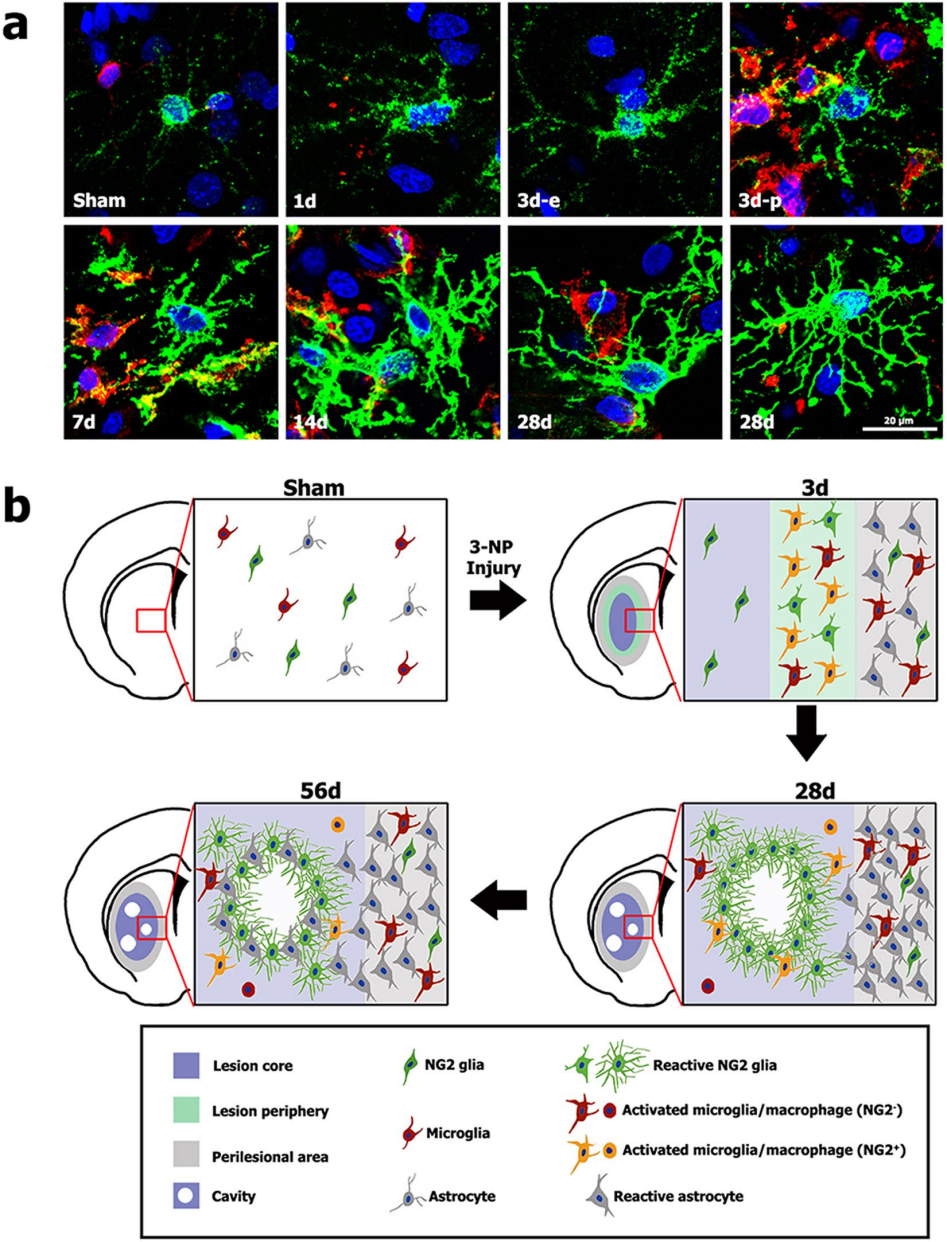
The morphological evolution of NG2 glia in striata treated with 3-NP (**a**) and schematic representation of dynamic NG2 glia in the lesion core and perilesional area (**b)**. (**a**) Double-labeling for NG2 and Iba1 showing that the morphologies of NG2 glia are dramatically altered with time, as thin and unbranched processes are transformed to thicker and highly ramified processes. Note that the processes of NG2 glia in the lesion periphery (3d-p), where activated microglia have infiltrated, show a more ramified morphology than those in the microglia-free epicenter (3d-e) 3 days post-lesion. Cell nuclei were stained with DAPI. (**b**) Three days after 3-NP injection, the lesion was progressively infiltrated with activated microglia migrating from the perilesional area. The accumulation of reactive NG2 glia and NG2-positive microglia was evident at the lesion periphery, although these cell types were absent from the lesion core, where resting NG2 glia were localized. In addition, the numbers of reactive astrocytes increased in the perilesional area, although these cells were absent from the lesion core. Twenty-eight days post-lesion, thick and highly branched processes of reactive NG2 glia form a cell network in the astrocyte-free lesion core primarily surrounded the cavities, while the astroglial scar had matured in the perilesional area. Fifty-six days post-lesion, NG2 glia were associated with astrocytes in the lesion core and the borders of the cavities.

In summary, here we describe the spatiotemporal pattern and phenotypic characterization of NG2 glia and the interactions among NG2 glia, astrocytes, and microglia in the lesion core in striata treated with 3-NP (Fig. 8). Our results further support the link between the transformation of NG2 glia to the reactive form and microglial activation/recruitment in response to brain insults. Our data also provide detailed information regarding the morphologies of NG2 glia in the lesion core, suggesting that NG2 glia may be involved in fibrotic scar formation in the lesion core, and later in glial scar formation in association with astrocytes.

## Methods

### Animals

All experimental procedures were conducted in accordance with the Laboratory Animal Welfare Act, the Guide for the Care and Use of Laboratory Animals, and Guidelines and Policies for Rodent Survival Surgery, and were approved by the Institutional Animal Care and Use Committee at the College of Medicine, The Catholic University of Korea (Approval Number: CUMC-2016-0014-02). All efforts were made to minimize animal suffering and to reduce the number of animals used.

Adult, male Sprague-Dawley rats (250–300 g, aged 9–11 weeks) were used in this study. Animals were housed in groups of three per cage and the animal holding room was maintained at a temperature of 22–25 °C and humidity of 50 ± 10% with food (gamma ray-sterilized diet) and water (autoclaved tap water) available *ad libitum*. They were maintained on a 12-h light/dark cycle. 3-NP (Sigma-Aldrich, St. Louis, MO, USA) was dissolved in buffered saline (pH = 7.0), and administered intraperitoneally at a dose of 15 mg/kg once daily for 3 days. All 3-NP-injected rats were evaluated daily for the presence of behavioral deficit, and only rats exhibiting neurological deficit symptoms, including hind limb impairment or kyphotic posture, recumbency, and impaired postural adjustments, were included in the experimental group^43^.

Animals were sacrificed 6 hours, 1, 2, 3, 7, 14, 28, and 56 days after the final injection of 3-NP (n = 3/time point). The control group (n = 3) received intraperitoneal injections of the same volume of normal saline for 3 consecutive days and were sacrificed 3 days after the final injection. The animals were perfused transcardially with 0.01 M phosphate buffered saline (pH 7.4) for 5 minutes followed by 4% paraformaldehyde in 0.1 M phosphate buffer (pH 7.4) for 30 minutes after being anesthetized with 10% chloral hydrate (4 mL/kg, intraperitoneal). Brains were dissected and post-fixed in the same fixative for 6 hours at 4 °C. The tissues were equilibrated with 30% sucrose in 0.1 M phosphate buffer and fully frozen.

### Immunohistochemistry

For NG2 immunohistochemistry, coronal cryostat sections (25-μm thick) were incubated in blocking buffer solution (3% normal goat serum, 0.05% Triton X-100 and 1% bovine serum albumin in phosphate-buffered saline) and then incubated overnight at 4 °C with a rabbit polyclonal antibody to NG2 (1:500; Millipore, Temecula, CA, USA). Primary antibody binding was visualized using peroxidase-labelled goat anti-rabbit antibody (1:100; Jackson ImmunoResearch, West Grove, PA, USA) and 0.05% 3,3′-diaminobenzidine tetrahydrochloride with 0.01% H_2_O_2_ as a substrate. The specificity of NG2 immunoreactivity was confirmed by the absence of immunohistochemical staining in sections from which the primary or secondary antibody had been omitted. Tissue sections were scanned and photographed using a slide scanner (SCN400, Leica Microsystems Ltd., Mannheim, Germany). Images were converted to TIFF format, and contrast levels adjusted using Adobe Photoshop v. 10.0 (Adobe Systems, San Jose, CA, USA).

For the evaluation of tissue injury, serial sections from rats at 3 days of survival after the last injection of 3-NP were processed for FJB histochemistry and immunohistochemistry for GFAP. For FJB staining, sections were stained with 0.0004% FJB (Millipore) in distilled water containing 0.01% acetic acid for 30 minutes according to the manufacturer’s protocol. After rinsing in distilled water, the sections were immersed in xylene and cover-slipped with the DPX mounting medium (Sigma-Aldrich).

For triple labeling, free floating sections (25-μm thick) were incubated with blocking buffer solution (0.2% gelatin, 1% bovine serum albumin and 0.05% saponin) and then with following two sets of antibodies at 4 °C overnight: the first included rabbit polyclonal antibodies to NG2 (1:500; Millipore), goat polyclonal antibody to Iba1 (1:300; Abcam, Cambridge, UK), and mouse monoclonal antibodies to GFAP (1:700; Millipore), and the second included rabbit polyclonal antibodies to NG2 (1:500; Millipore), goat polyclonal antibody to Iba1 (1:300; Abcam) and mouse monoclonal antibodies to ED1 (1:50; Bio-Rad, Hercules, CA, USA). In addition, double staining was performed using a mix of rabbit polyclonal antibodies against NG2 (1:500; Millipore), and one of following antibodies: biotin-conjugated isolectin B4 derived from Bandeiraea simplicifolia (1:50; Sigma-Aldrich) and mouse monoclonal antibody against RECA1 (1:200; Bio-Rad). Protein expression was detected using a Cy3-conjugated donkey anti-goat antibody (1:2000; Jackson ImmunoResearch), Cy3-conjugated streptavidin (1:1200; Jackson ImmunoResearch), Alexa Fluor 488-conjugated donkey anti-rabbit antibody (1:300; Thermo Fisher, Waltham, MA, USA), or Alexa Fluor 647-conjugated donkey anti-mouse antibody (1:300; Thermo Fisher). Negative staining controls for the triple immunofluorescence were performed by omission of the primary or secondary antibodies. In addition, we compared the results of triple labeling with those from single and double labeling of all combination of antibodies for the clear interpretation and avoidance of the cross reactivity. Counterstaining of cell nuclei was carried out using DAPI (4′,6-diamidino-2′-phenylindole; 1:2000; Roche, Mannheim, Germany) for 10 minutes. Slides were viewed with a confocal microscope (LSM 700; Carl Zeiss Co. Ltd., Oberkochen, Germany) equipped with four lasers (Diode 405, Argon 488, HeNe 555, and HeNe 639) under constant viewing conditions. Images were converted to TIFF format, and contrast levels and sizes were adjusted using Adobe Photoshop v.10.0.

To simultaneously detect apoptotic cells as well as NG2 expression and neuroglial activation, we performed double staining using TUNEL, according to the manufacturer’s protocol (Roche Diagnostics Corporation, Indianapolis, IN, USA), and one of following antibodies: goat polyclonal antibody against Iba1 (1:300; Abcam), mouse monoclonal antibody against GFAP (1:700; Millipore), mouse monoclonal antibody against NeuN (1:500; Millipore), and rabbit polyclonal antibodies against NG2 (1:500; Millipore). This was followed by a 1-h incubation with Cy3-conjugated streptavidin (1:1200; Jackson ImmunoResearch) for TUNEL staining, and one of following antibodies: Alexa Fluor 488-conjugated donkey anti-goat antibody (1:300; Thermo Fisher), Alexa Fluor 488-conjugated goat anti-mouse antibody (1:300; Thermo Fisher), and Alexa Fluor 488-conjugated goat anti-rabbit antibody (1:300; Thermo Fisher). Counterstaining of cell nuclei was carried out with DAPI for 10 min.

### Quantitative analysis

To quantify time-dependent changes in NG2^+^/Iba1^+^ and NG2^+^/Iba1^−^ cells in the striatum subjected to 3-NP, sections double-labeled for NG2 and Iba1 from control and experimental rats 1, 3, 7, and 14 days after 3-NP injection (n = 3 per time point) were obtained from the invariable region 0.20 mm to 1.20 mm dorsal to the bregma^77^. Five areas (160 × 160 μm per field) were chosen in the lesion core of each section and the corresponding striatum from control sections. Images from these areas were captured at 400 × magnification under constant viewing conditions. Because two areas of the lesion core, the lesion epicenter and periphery, were clearly distinguished by the presence of Iba1-positive microglia in the experimental rats 3 days after 3-NP injection, five areas were chosen from each of the two areas. NG2^+^/Iba1^+^ and NG2^+^/Iba1^−^ cells were counted only when their nuclei were clearly observed. The results of cell counts are presented as the mean ± standard error of the mean (SEM). Data analysis was performed using one-way analysis of variance followed by Bonferroni’s multiple comparisons test. Differences with *P* values less than 0.05 were considered significant. All statistical analyses were performed using GraphPad Prism version 5 (GraphPad Software Inc.; San Diego, CA).

### Quantification of proliferating NG2 glia

In order to label proliferating cells, rats (n = 3 per time point) were intraperitoneally injected with BrdU (Sigma-Aldrich, 50 mg/kg) at one of three time points, i.e., 2, 12, or 24 hours before being sacrificed on days 1 or 3 post-injection. Rats in the control group (n = 3) were also injected under the same conditions. For triple-immunofluorescence histochemistry, sections were pretreated to denature DNA and incubated with a rat monoclonal anti-BrdU antibody (1:200; Accurate Chemical & Scientific Corporation; Westbury, NY), a rabbit polyclonal antibody to NG2 (1:500; Millipore), and a goat polyclonal antibody to Iba1 (1:300; Abcam). The sections were then incubated in a mixture of Cy3-conjugated donkey anti-goat antibody (1:2,000; Jackson ImmunoResearch), Alexa Fluor 488-tagged goat anti-rabbit antibody (1:300; Thermo Fisher), and Cy5-conjugated anti-rat antibody (1:500; Abcam) for 2 hours at room temperature. Slides were viewed using a confocal microscope under constant viewing conditions.

Three coronal sections per animal were obtained from the invariable region 0.20 mm to 1.20 mm dorsal to the bregma^77^. Five areas (160 × 160 μm per field) in the lesion core were chosen and captured at 400× magnification using a confocal laser microscope. As described above, five areas each were chosen in the lesion periphery and the epicenter in sections from rats obtained 3 days post-lesion. The results of the cell counts are presented as the mean ± SEM. Data analysis was performed using analysis of variance followed by Bonferroni’s multiple comparisons test. Differences with *P* values less than 0.05 were considered significant. All statistical analyses were performed using GraphPad Prism version 5.

### Western blot analysis

For the immunoblot analysis, protein was isolated from the striatum of both controls and experimental rats at 7, 14, and 28 days after 3-NP injection. Samples were treated with boiling lysis buffer (1% sodium dodecyl sulfate, 1.0 mM sodium orthovanadate, 10 mM Tris, pH 7.4). Equal amounts (30 μg) of total protein were separated by sodium dodecyl sulfate polyacrylamide gel electrophoresis (10%) and transferred to polyvinylidene difluoride membranes. Immunostaining of the blots was performed using two primary antibodies, rabbit polyclonal antibody to NG2 (1:1000; Millipore) and mouse monoclonal antibody to anti-β-actin (1:10,000; Sigma-Aldrich). Membranes were then incubated with peroxidase-coupled secondary antibodies (1:2,000; Millipore) for 1 h at room temperature. Blots were developed using the Amersham ECL Prime western blotting detection reagent (GE healthcare, Little Chalfont, UK). Densitometric analysis was performed using the Eagle Eye TMII Still Video System (Stratagene, La Jolla, CA, USA). Statistical significance was determined by analysis of variance followed by Bonferroni’s multiple comparisons test. Differences with *P* values of less than 0.05 were considered significant. Three animals were used for immunoblotting at each time point, and relative optical densities of the protein bands were obtained from 3 independent experiments, each performed in triplicate.

### Immunoelectron microscopy and correlative light and electron microscopic study

For pre-embedding immunoelectron microscopy, floating vibratome sections (50-μm thick) from rats at 1, 5 and 28 days of survival after the last injection of 3-NP (*n* = 3) were immunostained with a rabbit polyclonal antibody to NG2 (1:500; Millipore). Immunoreactions were visualized with 3,3′-diaminobenzidine tetrahydrochloride as a chromogen. After post-fixation, dehydration and embedding in Epon 812, areas of interest were excised and glued onto resin blocks. After being cut into ultrathin sections (70–90 nm thick), they were observed in an electron microscope (JEM 1010, JEOL, Tokyo, Japan) with slight uranyl acetate staining.

For correlative light and electron microscopic study, vibratome sections (100 μm thick from rats at 5 days post-lesion were cryoprotected with 2.3 M sucrose in 0.1 M phosphate buffer and frozen in liquid nitrogen. Semithin cryosections 1 μm thick were cut at −100 °C with a glass knife in a Leica EM UC7 ultramicrotome equipped with the FC7 cryochamber (Leica, Wetzlar, Germany). The sections were double-labeled using a mix of rabbit polyclonal antibody to NG2 (1:500; Millipore) and goat polyclonal antibody to Iba1 (1:300; Abcam). Sections were labeled with DAPI for 10 min. Coverslipped sections were examined with a confocal microscope and photographed at various magnifications with a differential interference contrast setting to find specific areas for later examination by electron microscopy. After the coverslips had been floated off the sections, the tissues were prepared further for electron microscopy, as described above. To localize the immunofluorescent signals observed by light microscopy, contours within entire areas of interest were visualized with differential interference contrast microscopy, and the distinctive morphological profiles of cell nuclei or vascular profiles thus obtained were matched with their counterparts in the ultrathin sections.

## Electronic supplementary material

Supplementary information
