# Supplementary material for: Morphological characterization of NG2 glia and their association with neuroglial cells in the 3-nitropropionic acid–lesioned striatum of rat
Source: Sci Rep. 2018 Apr 13;8:5942. doi: 10.1038/s41598-018-24385-0 (PMC5899159; doi:10.1038/s41598-018-24385-0)
Supplement: Supplementary file 1 — Supplementary information [file 41598_2018_24385_MOESM1_ESM.pdf]

Supplementary information

Morphological characterization of NG2 glia and their association with neuroglial cells in the 3-nitropropionic acid–lesioned striatum of rat

Xuyan Jin[1], Tae-Ryong Riew[1], Hong Lim Kim[2], Jeong-Heon Choi[1], Mun-Yong Lee[1*]

[1]Department of Anatomy, Catholic Neuroscience Institute, College of Medicine, The Catholic University of Korea

[2]Integrative Research Support Center, Laboratory of Electron Microscope, College of Medicine, The Catholic University of Korea

[*]Corresponding Author: Mun-Yong Lee, E-mail: munylee@catholic.ac.kr

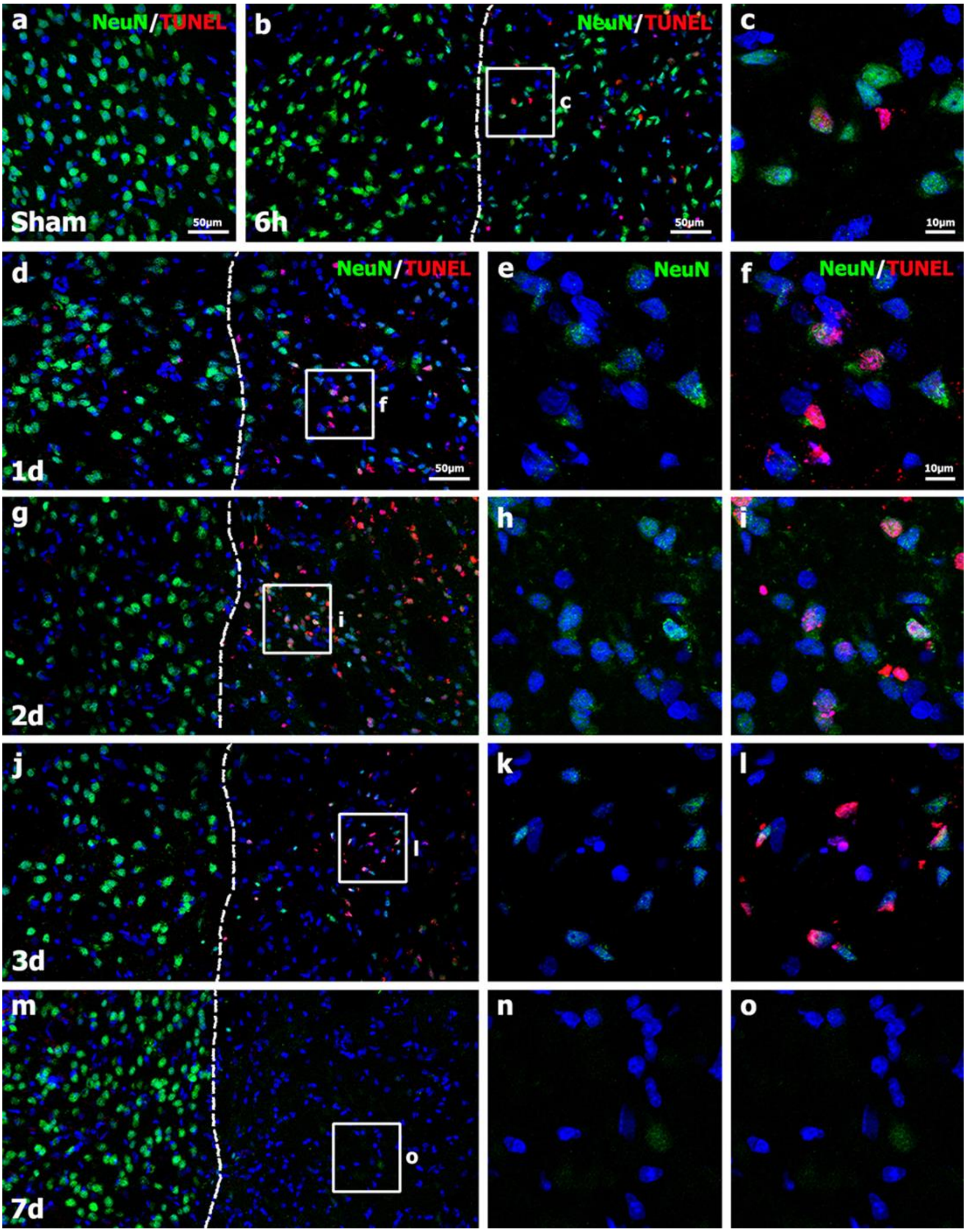
a
NeuN/TUNEL
Sham
50μm
b
NeuN/TUNEL
c
6h
50μm
c
10μm
d
NeuN/TUNEL
f
1d
50μm
e
NeuN
f
NeuN/TUNEL
10μm
g
i
2d
h
i
j
l
3d
k
l
m
o
7d
n
o

Supplementary Figure 1. Representative images showing the temporal profiles of neurodegeneration in the striata of rats treated with 3-NP. Neuronal nuclear marker (NeuN) and terminal deoxynucleotidyl transferase dUTP nick end labelling (TUNEL) double staining was performed on control and lesioned striatal sections. (a) No specific TUNEL staining was observed in the striatal neurons of saline-treated controls. (b and c) Six hours post-lesion, TUNEL staining was restricted to the lesion core (right side of the broken line), in which some neurons were found to be dying. (c) Higher-magnification view of the boxed area in b. (d–f). One day after 3-NP injection, TUNEL staining in the lesion core (right side of the broken line) appeared to be higher than that noted six hours post-lesion. Note that most of the TUNEL-positive cells were neurons. (g–i) Two days post-lesion, most of the remaining striatal neurons in the lesion core (right side of the broken line) were positive for TUNEL. (e, f, h, and i) Higher-magnification views of the boxed areas in d and g, respectively. (j–o) Lower- (j and m) and higher-magnification (k, l, n, and o) views of the NeuN/TUNEL double-labeled sections from brains obtained on days 3 (j–l) and 7 (m–o) post-lesion reveal that almost all striatal neurons had virtually disappeared from the lesion core, although some neuronal debris remained. Cell nuclei are stained with DAPI.

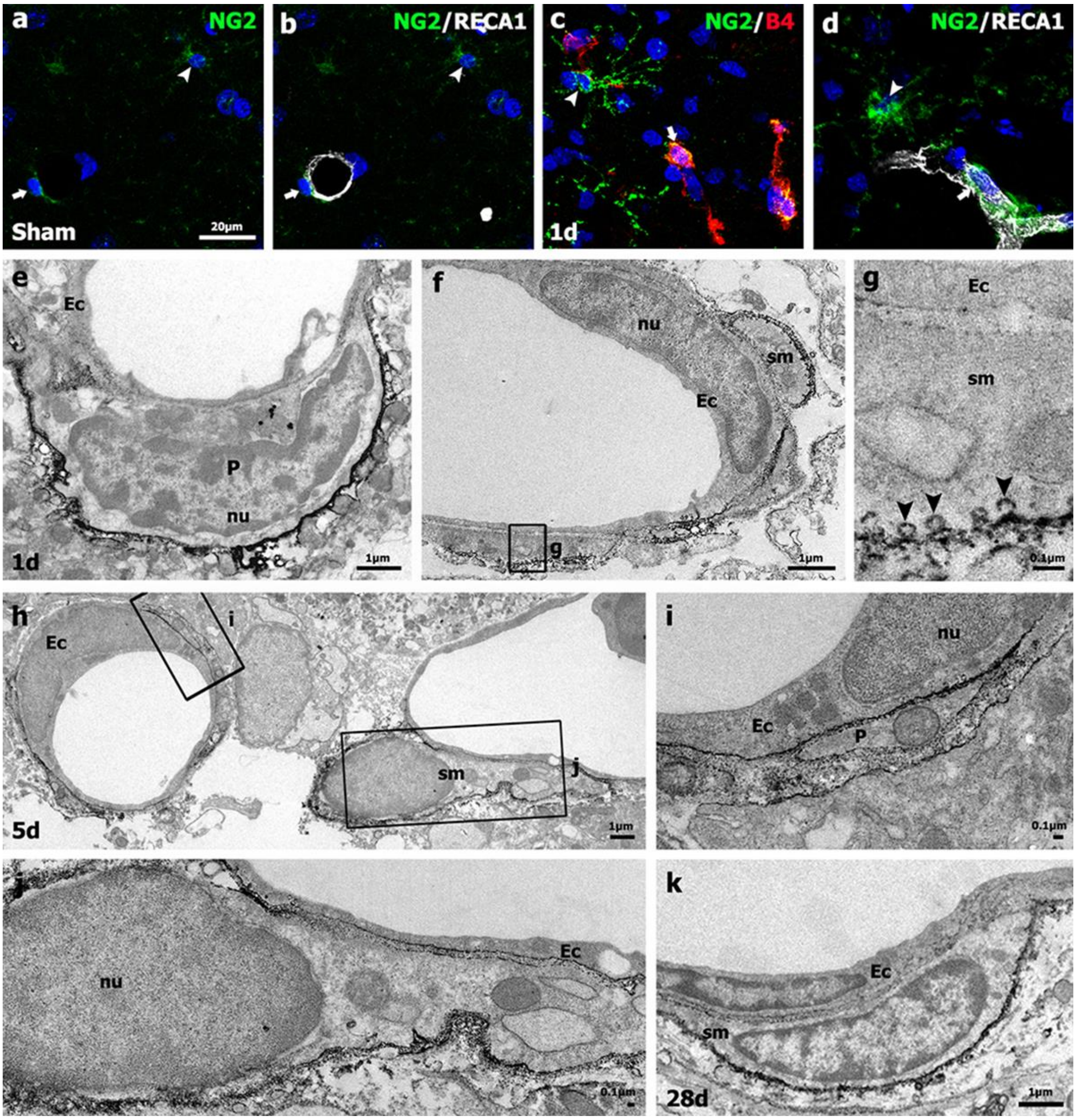
a
NG2
Sham
20μm
b
NG2/RECA1
c
NG2/B4
1d
d
NG2/RECA1
e
Ec
P
nu
1d
1μm
f
nu
sm
Ec
g
1μm
g
Ec
sm
0.1μm
h
Ec
i
sm
j
5d
1μm
i
nu
Ec
P
0.1μm
j
nu
Ec
0.1μm
k
Ec
sm
28d
1μm

Supplementary Figure 2. Characterization of NG2-positive cells associated with the vasculature in the lesion core. (a and b) Double-labeling for NG2 and vascular endothelial-cell marker RECA1 indicated the presence of weak NG2 expression in vascular cells (arrows), as well as in ramified NG2 glia (arrowheads) in control sections. (c and d) Double-labeling for NG2 and two vessel-associated markers, isolectin B4 (c) or RECA1 (d), one day post-lesion. Note that NG2 expression was observed within or in close proximity to the vasculature (arrows) and in cells with multiple fine processes (arrowheads) in the lesion core. (e–k) Ultrastructural characterization of vessel-associated NG2-positive cells in the lesion core on days 1, 5, and 28 post-lesion. (e–g) One day after 3-NP injection, NG2 immunoreac tivity was specifically observed in the plasma membrane and the adjacent extracellular matrix of the pericytes (P) and smooth muscle cells (sm), but not in endothelial cells (Ec). (g) Higher-magnification view of the boxed areas in f. Arrowheads in g point to the plas ma membrane caveolae of smooth muscle cells. (h–j) Immuno-electron microscopic images obtained on day 5 post-lesion indicated that NG2 was specifically localized along the plasma membrane of pericytes (P) and smooth muscle cells (sm), both of which were closely apposed to endothelial cells (Ec). Note that endothelial cells were devoid of NG2. (i and j) H igher-magnification views of the boxed areas in h. (k) On day 28 post-lesion, electron-dense grains indicative of NG2 expression were specifically localized along the plasma mem brane of a smooth muscle cell (sm). Note that the endothelial cell (Ec) was devoid of NG2 expression. nu: nuclei. Cell nuclei are stained with DAPI
